# ﻿Revision of *Sphenoraia* Clark, 1865 (Coleoptera, Chrysomelidae, Galerucinae) from China, with descriptions of two new species

**DOI:** 10.3897/zookeys.1132.89858

**Published:** 2022-11-25

**Authors:** Chuan Feng, Xing-Ke Yang, Zhi-Qiang Li, Yang Liu

**Affiliations:** 1 Key Laboratory of Resource Biology and Biotechnology in Western China, Northwest University, Taibai North Road 229, Xi’an 710069, China; 2 Shaanxi Key Laboratory for Animal Conservation, College of Life Science, Northwest University, Taibai North Road 229, Xi’an 710069, China; 3 Key Laboratory of Zoological Systematics and Evolution, Institute of Zoology, Chinese Academy of Sciences, Beijing 100101, China; 4 Guangdong Public Laboratory of Wild Animal Conservation and Utilization, Institute of Zoology, Guangdong Academy of Sciences Guangzhou, Guangdong 510260, China; 5 Guangdong Key Laboratory of Animal Conservation and Resource Utilization, Institute of Zoology, Guangdong Academy of Sciences, Guangzhou, Guangdong 510260, China

**Keywords:** Leaf beetles, new combination, new species, *Sphenoraia*, taxonomy

## Abstract

In this study, ten species of *Sphenoraia* Clack, 1865 are recognized and re-described: Sphenoraia (Sphenoraioides) anjiensis Yang & Li, 1998, Sphenoraia (Sphenoraioides) berberii Jiang, 1992, Sphenoraia (Sphenoraioides) duvivieri (Laboissière, 1925), Sphenoraia (Sphenoraioides) haizhuensis Yang, 2021, Sphenoraia (Sphenoraioides) micans (Fairmaire, 1888), Sphenoraia (Sphenoraioides) nebulosa (Gyllenhal, 1808), Sphenoraia (Sphenoraioides) nigromaculata Jiang, 1992, Sphenoraia (Sphenoraioides) punctipennis Jiang, 1992, Sphenoraia (Sphenoraioides) rutilans (Hope, 1831), and Sphenoraia (Sphenoraioides) yajiangensis Jiang, 1992. Two new species, Sphenoraia (Sphenoraia) decemmaculata Feng, Yang & Liu, **sp. nov.** and Sphenoraia (Sphenoraioides) flavomarginata Feng, Yang & Li, **sp. nov.**, are described. Additionally, Sphenoraia (Sphenoraia) cupreata Jacoby, 1890 and Sphenoraia (Sphenoraia) nigra Wang, Li & Yang, 2000 are transferred from *Sphenoraia* to *Gallerucida*. A key to the 12 Chinese species of *Sphenoraia* is given.

## ﻿Introduction

*Sphenoraia* was established by Clark (1865), with *Gallerucabicolor* (Hope, 1831) as the type species. Sphenoraia is the senior synonym of the subgenus Neosermylassa Chûjô, 1956 synonymized by Kimoto (1986). All known species are distributed in the Palearctic and Oriental regions. Currently, there are 25 known species of *Sphenoraia* worldwide, among which 12 species occur in China (Yang et al. 1998; Wang et al. 2000; Nie et al. 2017). In this paper we describe the main generic characters of *Sphenoraia* according to those defined by Wang et al. (2000) based on examination of type material for most species. The species of this genus can be distinguished by the following characters: head small, frontal tubercle distinct, antennae slender and extend to the middle of each elytron, antennomere 2 shortest, antennomere 3 nearly equal in length and shape to antennomere 2 or slightly longer than 2, antennomere 4 longest and longer than antennomeres 2 and 3 combined. The pronotum is wider than the head, being nearly twice as broad as it is long, basal and with the apical border not margined, the lateral border margined; the disc of pronotum is without deep depression. Scutellum triangular, smooth, normally impunctate. The elytra are broader at the base than where they join the pronotum, the humeri are strongly convex, the disc is strongly raised and has punctures. The elytral epipleuron is broad at the base, and gradually narrows from its center, extending to the apex of the elytron. The procoxal cavity is closed behind, and the procoxa is globose. Claws are appendiculate, with a sclerotized appendage underneath. Male with apex of last visible sternite trilobed; female with the last visible sternite complete (Jiang 1992; Wang et al. 2000). This genus can be divided into two subgenera, *Sphenoraioides* and *Sphenoraia*, according to the shape of the body and antenna type (Yang et al. 2015).

## ﻿Materials and methods

The morphological characters were examined with an Olympus SZ61 microscope. The genitalia of males from each species were dissected using the following procedure: for dried or ethanol preserved specimens, the abdomen was removed from each specimen, bathed in boiling water for 5–10 minutes, then transferred to a vial containing 10% KOH solution. The abdomen with the aedeagus was washed in distilled water several times, transferred onto a cavity slide using fine forceps and the aedeagus was separated from the abdomen using a hooked, fine dissecting needle.

Habitus images were taken using a Canon 5DSR/Nikon SMZ25 digital camera. Aedeagus images were taken using a Nikon D610 digital camera, attached to a Zeiss V/A1 microscope (with 5× objective lens). A cable shutter release was used to prevent the camera from shaking. To obtain the full depth of focus, all images were stacked using HELICON FOCUS 7 and the resulting output was edited with Adobe Photoshop CC.

The material in this study is deposited in the following institutions: **GDAS** Institute of Zoology, Guangdong Academy of Sciences, Guangzhou, China and **IZAS**Institute of Zoology, Chinese Academy of Sciences, Beijing, China

## ﻿Results

*Sphenoraia* is similar to several genera, and a short key to the more closely related genera of *Sphenoraia* in the large subfamily Galerucinae is provided below.

### ﻿Key to the similar genera of Hylaspini

**Table:** 

1	Anterior metasternal process not extending beyond the front edge of the meso-coxal cavities, basal border of pronotum not margined	**2**
–	Anterior metasternal process extending beyond the front edge of the meso-coxal cavities, pronotum borders margined, pronotum with a pair of transverse depressions or without depressions	***Gallerucida* Motschulsky, 1860**
2	Anterior and lateral border of pronotum margined, posterior corner of pronotum acute, disc with deep transverse depressions	***Aplosonyx* Chevrolat, 1837**
–	Lateral border of pronotum margined, posterior corner of pronotum rounded, disc without deep transverse depressions	***Sphenoraia* Clark, 1865**

#### ﻿ Sphenoraia

Taxon classificationAnimaliaColeopteraChrysomelidae

Clark, 1865

AB29A81C-0D81-5CBB-BA3D-433066ABA8FC

Sphenoraia Clark, 1865: 257, 262. Type species: Gallerucabicolor Hope, 1831, designated by Gressitt and Kimoto 1963.
Sermylassa subgenus Neosermylassa Chujo, 1956: 14. Type species: Semylassa (Neosermylassa) japonica Chûjô, 1956, by monotypy and original designation. Synonymized by Kimoto 1986: 312.

### ﻿Key to the Chinese species of *Sphenoraia*

**Table:** 

1	Body shape elliptical; antennae short, antennomere 3 almost as long as antennomere 2 in male, from antennomere 4 they become gradually broader [Sphenoraia (Sphenoraioides)]	**2**
–	Body shape nearly parallel; antennae filiform, antennomere 2 shorter than 3 in male, several antennomeres of apex slightly thick [Sphenoraia (Sphenoraia)]. Head and pronotum yellowish brown, pronotum without any black spots, antennae and legs black	**Sphenoraia (Sphenoraia) decemmaculata sp. nov.**
2	Head and pronotum yellow or yellowish brown, pronotum usually with one pair of black spots, elytron with seven black spots	**3**
–	Head and pronotum dark green or bluish black	**4**
3	Scutellum without distinct punctures	**Sphenoraia (Sphenoraioides) nebulosa (Gyllenhal, 1808)**
–	Head and scutellum with distinct punctures	**Sphenoraia (Sphenoraioides) haizhuensis Yang, 2021**
4	Elytra without any spots or stripes	**5**
–	Elytra with black spots or yellow stripes	**7**
5	Elytra reddish brown	**Sphenoraia (Sphenoraioides) duvivievi (Laboissière, 1925)**
–	Elytra not reddish brown	**6**
6	Elytra bluish green or red	**Sphenoraia (Sphenoraioides) micans (Fairmaire, 1888)**
–	Elytra bluish black; pronotum with a pair of shallow depressions laterally	**Sphenoraia (Sphenoraioides) rutilans (Hope, 1831)**
7	Elytra yellow, each elytron with five black spots	**Sphenoraia (Sphenoraioides) anjiensis Yang & Li, 1998**
–	Elytra blackish green, with yellow stripes	**8**
8	Epipleuron yellow, elytra surface without any spots or stripes	**Sphenoraia (Sphenoraioides) yajiangensis Jiang, 1992**
–	Epipleuron yellow, elytra surface with stripes	**9**
9	Elytra surface with one transverse yellow stripe at subapex	**Sphenoraia (Sphenoraioides) flavomarginata sp. nov.**
–	Elytra with yellow stripes at base and apex	**10**
10	Stripes at base and apex not joined at the middle suture	**Sphenoraia (Sphenoraioides) berberii Jiang, 1992**
–	Stripes at base and apex joined in middle suture	**11**
11	Elytra divided into four parts by stripes	**Sphenoraia (Sphenoraioides) punctipennis Jiang, 1992**
–	Elytra divided into seven parts by stripes	**Sphenoraia (Sphenoraioides) nigromaculata Jiang, 1992**

#### ﻿ Sphenoraia (Sphenoraioides) anjiensis

Taxon classificationAnimaliaColeopteraChrysomelidae

Yang & Li, 1998

F46BBD8A-C217-50D1-B9F8-5F028032CEBA

Fig. 1A–F

Sphenoraia (Sphenoraioides) anjiensis Yang & Li, 1998: 132.

##### Type specimens examined.

***Holotype***: ♀, China, Zhejiang Province, Anji, Longwang Mountain; 1500 m; 13 May 1996; Hong Wu leg.; IZAS. ***Paratype***: 1♂ China, Zhejiang Province, Anji, Longwang Mountain; 26 Jul. 1996; Hong Wu leg.; IZAS.

##### Additional specimen examined.

1♀, China, T`ienmll Shan (Tianmu Mountian), Musèe Heude; 21 Jul. 1936; D. Piel. leg.; IZAS.

##### Description.

**Male.** Length 7.2 mm, width 4.6 mm.

Head, antennae, pronotum, ventral surface of thorax, scutellum, and legs black, elytra and abdomen yellow; each elytron with five black spots, base with one pair of spots and apex with one spot, median with a large transverse band and subapex with a large spot; abdomen with four pair of round black spots at side on the first, second, third, fourth visible sternites.

Vertex covered with punctures finely and sparsely; frontal tubercle distinctly raised, each separated by a deep furrow; antennae slender, extended to the middle of the elytra; antennomeres 1–3 thin, shiny; antennomeres 4–11 wide and flat, with short hairs, antennomere 4 approximately twice as long as it is wide; antennomeres 5–10 each approximately 1.6 × as long as they are wide; antennomere 2 shortest, antennomere 3 slightly longer than 2, 1.5 × as long as antennomere 2; antennomere 4 longest, 1.2 × as long as antennomeres 2 and 3 combined; antennomeres 5–10 gradually shortened, shorter than 4; antennomere 11 slightly longer than 10, pointed.

Pronotum approximately 1.9 × as wide as it is long, with lateral margins straight and parallel slightly, anterior angle thickened, produced forward, disc slightly convex, sparsely covered with small punctures.

Scutellum triangular, with rounded apex, smooth, impunctate.

Bases of both elytra combined wider than the pronotum, gradually widen posteriorly and rounded at the apexes; dorsal surface slightly convex and covered with large and deep regular punctures, partly arranged in ten rows on each elytron, the interstices of the punctures equal to the diameter of the punctures.

Metasternum twice as long as the mesosternum; prothoracic legs shortest, mesothoracic legs slightly longer, metathoracic legs longest.

Ventral surface of the abdomen with five segments, segment 1 longest, segments 2–4 gradually shortened, apical segment slightly longer than segment 4, with three lobes.

Aedeagus slender, parallel-sided, basally widened with triangular apex, distinctly pointed. In lateral view moderately bent.

**Female.** Length 7.0–7.2 mm, width 4.8–5.0 mm.

Antennae slender, antennomere 2 shortest, antennomere 3 slightly longer than 2, 1.4 × as long as second; antennomere 4 longest, longer than antennomeres 2 and 3 combined slightly; antennomeres 5–10 equal in length, shorter than 4; apical sternite flatted.

##### Differential diagnosis.

This species can be distinguished from other species by its black pronotum and the black spots on the abdomen.

##### Distribution.

China: Zhejiang.

**Figure 1.**
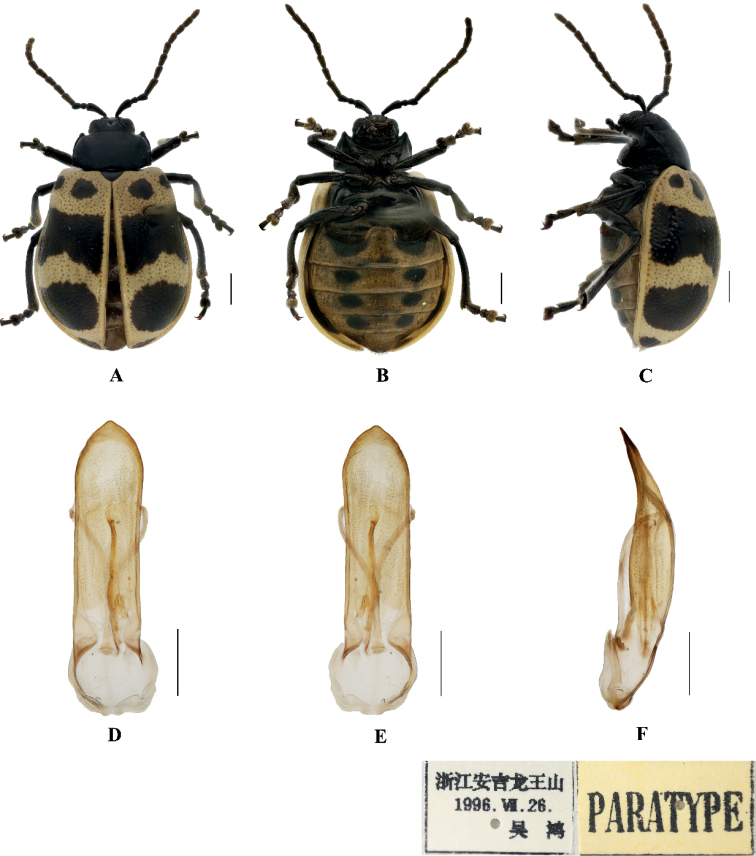
Sphenoraia (Sphenoraioides) anjiensis**A–C** habitus **D–F** aedeagus **A, D** dorsal views **B, E** ventral views **C, F** lateral views. Scale bars: 1 mm (**A–C**); 0.5 mm (**D–F**).

#### ﻿ Sphenoraia (Sphenoraioides) berberii

Taxon classificationAnimaliaColeopteraChrysomelidae

Jiang, 1992

B716D26E-090B-5B4E-B03A-6E82B7C1FCC5

Fig. 2A–F

Sphenoraia berberii Jiang, 1992: 665.
Sphenoraia (Sphenoraioides) berberii : Wang et al. 2000: 118.

##### Type specimens examined.

***Holotype***: ♂, China, Yunnan Province, Deqin, Baimang snowy mountain; 3300 m; 28 Aug. 1987; Shuyong Wang leg.; IZAS. ***Paratypes***: 10♂♂6♀♀, same information as holotype. ***Allotype***: 1♀, same information as holotype.

##### Additional specimen examined.

1♀, China, Yunnan Province, Lijiang, Yulong Mountain; 3200 m; 17 Jul. 1984; Jianguo Fan leg. IZAS.

##### Description.

**Male.** Length 5.9–6.4 mm, width 3.4–3.6 mm.

Head, pronotum, and scutellum blackish green, antennae, elytra, legs, and ventral surface of the body brown; apex of each abdominal segment yellow, elytral epipleuron from base to subapex yellow, connecting with yellow stripes on the base and apex of the elytra.

Vertex covered with punctures finely and sparsely; frontal tubercle distinctly raised, separated from each other by a deep furrow; antennae short, robust, extended to the middle of the elytra; antennomeres 1–3 thin, shiny; antennomeres 4–11 wide and flat, with short hairs, antennomeres 2 and 3 shortest, antennomere 3 nearly equal in length and shape to antennomere 2, antennomere 4 longest, 1.5 × as long as antennomeres 2 and 3 combined; antennomeres 5–10 gradually shortened, shorter than 4; antennomere 11 slightly longer than 10, pointed.

Pronotum approximately 1.8 × as wide as long, with lateral margins rounded, disc slightly convex, sparsely covered with punctures in the center, base, and apex of pronotum covered with punctures closely. The interstices between punctures equal to the diameter of each puncture.

Scutellum triangular, only on the base and apex, sparsely covered with punctures.

Bases of both elytra wider than pronotum, gradually widen posteriorly and rounded at apex; dorsal surface slightly convex, irregularly covered with punctures, the interstices between punctures equal to the diameter of individual punctures.

Metasternum twice as long as mesosternum; prothoracic legs shortest, mesothoracic legs slightly longer, metathoracic legs longest.

Ventral surface of abdomen with five segments, segment 1 longest, segments 2–4 gradually shortened, apical segment slightly longer than segment 4, with three lobes.

Aedeagus slender, rounded laterally, basally widened, with triangular apex, distinctly pointed. In lateral view moderately bent.

**Female.** Length 5.8–6.2 mm, width 3.5–3.8 mm

Antennae slender, antennomere 2 shortest, antennomere 3 slightly longer than 2, 1.2 × as long as second; antennomere 4 longest, twice as long as antennomeres 2 and 3 combined; apical sternite flatted.

##### Differential diagnosis.

This species can be distinguished from other species by its blackish green pronotum and the yellow stripes on the elytra.

**Figure 2.**
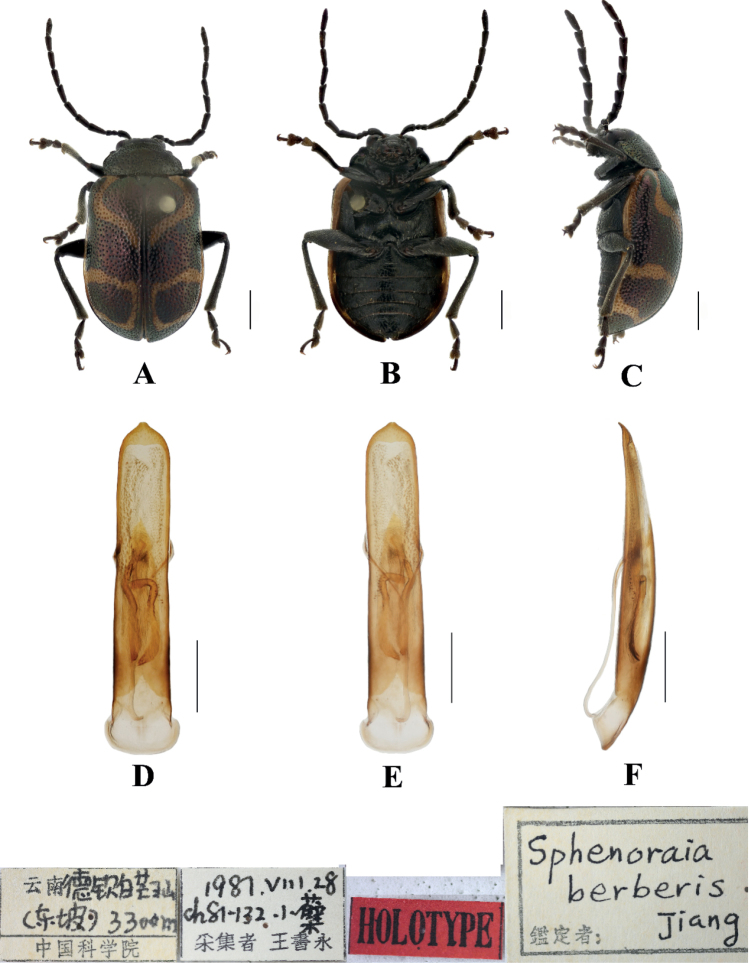
Sphenoraia (Sphenoraioides) berberii**A–C** habitus **D–F** aedeagus **A, D** dorsal views **B, E** ventral views **C, F** lateral views. Scale bars 1 mm (**A**); 0.5 mm (**D–F**).

##### Distribution.

China: Yunnan.

##### Host plant.

*Berberis* sp.

#### ﻿ Sphenoraia (Sphenoraioides) duvivieri

Taxon classificationAnimaliaColeopteraChrysomelidae

(Laboissière, 1925)

69F00801-2F3D-5333-94A2-61D4994AC083

Fig. 3A–F

Sphenoraia indica Duvivier, 1887: 48 (nec. Harold 1880).
Galerucida duvivier Laboissière, 1925: 53 (replacement name for Sphenoraiaindica Duvivier, 1887).
Sphenoraia (Sphenoraioides) duvivieri : Laboissière 1934: 134.
Gallerucida amala Maulik, 1936: 549. Synonymized by Laboissière 1940: 30.

##### Additional specimens examined.

2♂♂, China, Guangdong Province, Enping, Qixingkeng; 100 m; 21 Jun. 2022; Chuan Feng leg.; GDAS. 1♀, China, Guangxi Province, Napo, Beidou; 550 m; 11 Apr. 1998; Tianshan Li leg.; IZAS. 1♂, China, Guangxi Province, Longsheng, Baiyan; 1150 m; 21 Jun. 1963; Yongshan Shi leg.; IZAS. 1♀, China, Guangxi Province, Longzhou; 360 m; 20 Jun. 1963; Yongshan Shi leg.; IZAS. 1♂, China, Guangxi Province, Diding; 1000–1700 m; 23 Jun. 2000; Jian Yao leg.; IZAS. 1♂, China, Guangxi Province, Jinxiu, Shengtang Mountain; 900 m; 17 May 1999; Xuezhong Zhang leg.; IZAS. 1♂, China, Sichuan Province, Youyang; 9 Jul. 1989; Dazhi Dong leg.; IZAS. 1♂, China, Sichuan Province, Youyang; 9 Jul. 1989; Su Lin leg.; IZAS. 1♂, China, Guizhou Province, Guiyang; May–Jul. 1981; IZAS. 1♀, China, Guizhou Province, Maolan; 30 May 1998; Qiongzhang Song leg.; IZAS. 1♀, China, Yunnan Province, Funing; 250 m; 17 Apr. 1998; Chunsheng Wu leg.; IZAS. 2♂♂1♀, Yunnan Province, Xishuangbanna,Yunjinghong; 900 m; 27 Apr. 1958; Yiran Zhang leg.; IZAS. 1♀, China, Yunnan Province, Changning; 1700 m; 16 Jun. 1979; IZAS. 1♀, Yunnan Province, Xishuangbanna, Menglun; 600 m; 11 Sep. 1993; Huanli Xu leg.; IZAS.

##### Description.

**Male.** Length 6.8–7.8 mm, width 4.8–5.6 mm.

Head, antennae, pronotum, scutellum, legs, and ventral surface of thorax dark blue, elytra and abdomen brown.

Vertex finely and sparsely covered with punctures; frontal tubercle distinctly raised, separated from each other by a deep furrow; antennae short, robust, extended to the middle of elytra; antennomeres 1–3 thin, shiny; antennomeres 4–11 wide and flat, with short hairs, antennomere 4 approximately 2.2 × as long as wide; antennomere 5 approximately 1.8 × as long as wide; antennomeres 6 and 7 each approximately 1.5 × as long as wide; antennomeres 8–10, each approximately 1.2 × as long as wide; antennomere 11 approximately 1.5 × as long as wide; antennomere 2 shortest, antennomere 3 longer than 2 slightly, 1.2 × as long as second; antennomere 4 longest, 1.2 × as long as antennomeres 2 and 3 combined; antennomeres 5–10 differ in length, shorter than 4; antennomere 11 slightly longer than 10, pointed.

Pronotum approximately 2.7 × as wide as long, with rounded lateral margins; disc sparsely covered with punctures, with a lateral pair of shallow impressions.

Scutellum triangular, sparsely covered with punctures.

Bases of both elytra wider than pronotum, gradually widen posteriorly and rounded at apex; dorsal surface slightly convex, irregularly covered with punctures, the interstices between punctures wider than diameter of individual punctures, 1.5 × as wide as the diameter of punctures.

Metasternum twice as long as mesosternum; prothoracic legs shortest, mesothoracic legs slightly longer, metathoracic legs longest.

Ventral surface of abdomen with 5 segments, segment 1 longest, segments 2–4 gradually shortened, apical segment equal in length to segment 1, with three lobes.

Aedeagus slender, parallel-sided, basally widened, with rounded apex. In lateral view, strongly bent.

**Female.** Length 6.8–7.8 mm, width 4.8–5.6 mm.

Antennae antennomeres 1–5 thin, antennomeres 6–11 wide and flat, each approximately 1.4 × as long as wide; antennomere 2 shortest, antennomere 3 longer than 2 slightly, 1.5 × as long as second; antennomere 4 longest, slightly longer than antennomeres 2 and 3 combined; apical sternite flatted.

##### Differential diagnosis.

This species can be distinguished from other species in the genus by the mottled brown color of the body and shallow impressions of pronotum.

##### Distribution.

China: Hunan, Hong Kong, Guangdong, Guangxi, Guizhou, Sichuan Yunnan; Vietnam, Laos, Thailand, India, and Myanmar.

**Figure 3.**
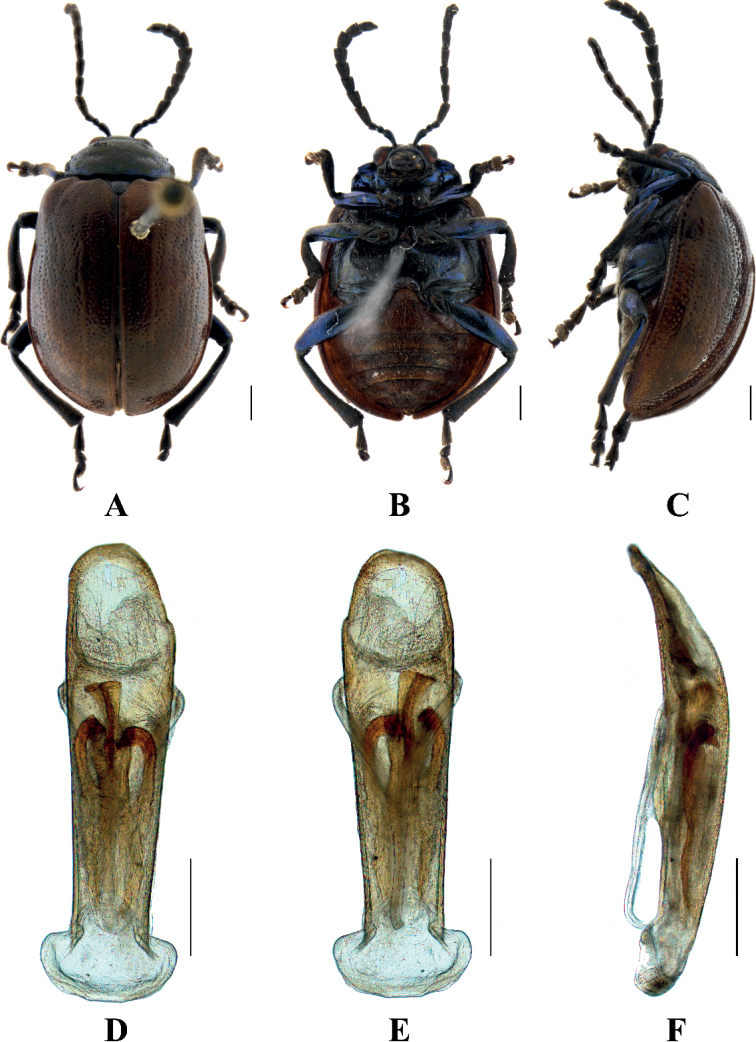
Sphenoraia (Sphenoraioides) duvivieri**A–C** habitus **D–F** aedeagus **A, D** dorsal views **B, E** ventral views **C, F** lateral views. Scale bars: 1 mm (**A–C**); 0.5 mm (**D–F**).

#### ﻿ Sphenoraia (Sphenoraioides) haizhuensis

Taxon classificationAnimaliaColeopteraChrysomelidae

Yang, 2021

2190C765-5986-5631-9033-51CBE50B3D30

Fig. 4A–E

Sphenoraia (Sphenoraioides) haizhuensis Yang, 2021: 245.

##### Type specimens examined.

***Holotype***: ♂, Guangdong Province, Guangzhou, Haizhu wetland; 113°18'24"E, 23°4'32"N; 20–23 May 2021; FIT-1; GDAS. ***Paratype***: 1♂, Guangdong Province, Guangzhou, Haizhu wetland; 113°21'29"E, 23°2'58"N; 2020.9.21–10.19; MT-9; GDAS.

##### Description.

**Male.** Length. 6.2–6.4 mm, width 4.0–4.2 mm.

Head, pronotum, elytra, and legs yellow, antennae and ventral surface of body yellowish brown, scutellum brown; pronotum with a black spot on each side; each elytron with seven black spots, basal, middle and subapex with one pair of spots and apex with one spot.

Vertex finely and sparsely covered with punctures; frontal tubercles distinctly raised and separated from each other by a deep furrow; antennae short, robust, extended to the middle of elytra; antennomeres 1–3 thin, shiny; antennomeres 4–11 wide and flat, with short hairs, segment 4 approximately twice as long as wide; antennomeres 5–10, each approximately 1.6 × as long as wide; antennomeres 2 and 3 shortest, antennomere 3 nearly equal in length and shape to antennomere 2, antennomere 4 longest, twice as long as antennomeres 2 and 3 combined; antennomeres 5–10 gradually shortened, shorter than 4; antennomere 11 slightly longer than 10, pointed.

Pronotum approximately 2.5 × as wide as long, with rounded lateral margins; disc slightly depressed on each side, sparsely covered with small punctures, with the punctures on pronotum larger than those on the head.

Scutellum triangular, sparsely covered with small punctures.

Base of both elytra wider than pronotum, gradually widen posteriorly and rounded at apex; dorsal surface slightly convex, irregularly covered with large and deep punctures, the interstices between punctures slightly wider than diameter of individual punctures.

Metasternum 2.5 × as long as mesosternum; prothoracic legs shortest, mesothoracic legs slightly longer, metathoracic legs longest.

Ventral surface of abdomen with five segments, segment 1 longest, segments 2–4 gradually shortened, apical segment slightly longer than segment 4, three lobes.

Aedeagus slender, parallel-sided, basally widened, apex rounded. In lateral view strongly bent.

##### Differential diagnosis.

This species can be distinguished from other species by black spots on the elytra. This species closely resembles Sphenoraia (Sphenoraioides) nebulosa, but the latter is without punctures in the scutellum.

##### Distribution.

China: Guangdong.

**Figure 4.**
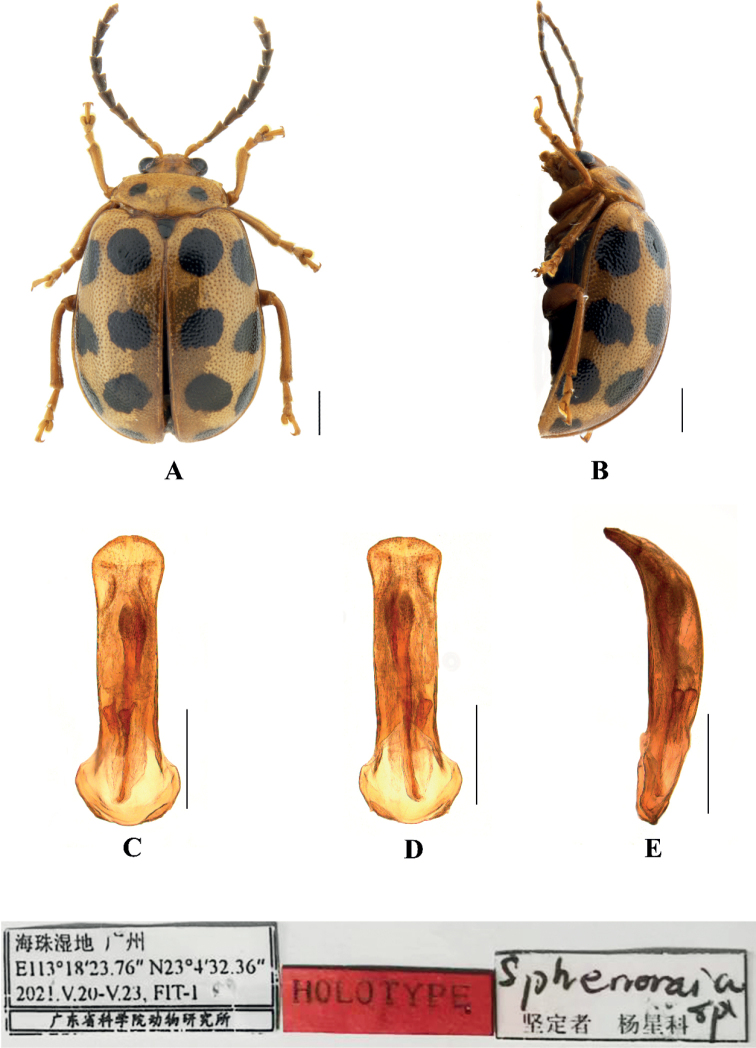
Sphenoraia (Sphenoraioides) haizhuensis**A, B** habitus **C–E** aedeagus **A, C** dorsal views **D** ventral views **B, E** lateral views. Scale bars: 1 mm (**A, B**); 0.5 mm (**C–E**).

#### ﻿ Sphenoraia (Sphenoraioides) micans

Taxon classificationAnimaliaColeopteraChrysomelidae

(Fairmaire, 1888)

07C21A20-8C72-5616-93C6-2A9015BED66B

Fig. 5A–F

Eustetha micans Fairmaire, 1888: 42.
Galerucida fulgida var. coerulescens Weise, 1922: 91. Synonymized by Wilcox 1971: 198.
Sphenoraia (Sphenoraioides) micans : Laboissière 1934: 131.
Sphenoraia (Sphenoraioides) micans var. cyanella Laboissière, 1934: 132. Synonymized by Wilcox 1971: 198.

##### Other specimens examined.

1♀, China, Henan Province, Luanshan; 1000 m; 10 Jul. 1996; Wanzhi Cai leg.; IZAS. 1♂, China, Henan Province, Luanshan; 1000 m; 10 Jul. 1996; Jikun Yang leg.; IZAS. 1♂1♀, China, Zhejiang Province, Anji, Longwang Mountain; 500 m; 12 Jun. 1996; Xingke Yang leg.; IZAS. 1♂, China, Zhejiang Province, Anji, Longwang Mountain; 14 Jun. 1996; 1400 m; Hong Wu leg.; IZAS. 1♀, China, Zhejiang Province, Tianmu Mountain; Jul. 2000; IZAS. 1♂ China, Zhejiang Province, Tianmu Mountain; Jul. 1999; IZAS. 1♂, China, Zhejiang Province, Tianmu Mountain; 600–800 m; 7 Jun. 1998; Mingyuan Gao leg.; IZAS. 1♀, China, Jiangxi Province, Jinggangshan, xiangzhou; 26 Apr. 2011; Yan Mei leg.; GDAS. 1♀, China, Hunan Province, Sangzhi, Tianpingshan; 700–1450 m; 14 Aug. 1988; Shuyong Wang leg. IZAS. 1♂, China, Wuyi Mountain; 22 Apr. 1997; Yanyu Wu leg.; IZAS. 1♀, China, Fujian Province, Guadangling; 29 Aug. 1983; Jiang Wang leg.; IZAS. 1♂, China, Guangxi Province, Longsheng, Hongtan; 900 m; 14 Jun. 1963; Shuyong Wang leg.; IZAS. 1♀, China, Guizhou Province, Chiqian; 670 m; 24 Jul. 1988; Shuyong Wang leg.; IZAS. 1♀, China, Guizhou Province, Fanjing Mountain; 2 Aug. 1988; Yongkun Li leg.; IZAS.

##### Description.

**Male.** Length 7.7–8.4 mm, width 5.2–5.8 mm.

Head, pronotum and elytra green, antennae, scutellum, legs, and ventral surface of thorax dark blue, abdomen yellowish brown. Some individuals with blue or red head, pronotum, and elytra.

Vertex finely and sparsely covered with punctures; frontal tubercle distinctly raised, separated from each other by a deep furrow; antennae short, robust, extended to the middle of elytra; antennomeres 1–3 thin, shiny; antennomeres 4–11 wide and flat, with short hairs, antennomere 4 approximately twice as long as wide; antennomeres 5–11, each approximately 1.5 × as long as wide; antennomere 2 shortest, antennomere 3 slightly longer than 2, 1.2 × as long as second; antennomere 4 longest, 1.2 × as long as antennomeres 2 and 3 combined; antennomeres 5–10 unequal in length, shorter than 4; antennomere 11 slightly longer than 10, pointed.

Pronotum approximately 2 × as wide as long, with rounded lateral margins; disc slightly convex, sparsely covered with punctures.

Scutellum triangular, with rounded apex, smooth, impunctate.

Bases of both elytra wider than pronotum, gradually widen posteriorly and rounded at apex; dorsal surface slightly convex, irregularly covered with punctures, densely covered with large punctures on humeral angle and sparsely covered in small punctures on other parts. The interstices between punctures wider than diameter of individual punctures on apex of the elytra, 2 × as wide as the diameter of individual punctures.

Metasternum 2.5 × as long as mesosternum; prothoracic legs shortest, mesothoracic legs slightly longer, metathoracic legs longest.

Ventral surface of abdomen with five segments, segment 1 longest, segments 2–4 gradually shortened, apical segment slightly longer than segment 4, with three lobes.

Aedeagus slender, parallel-sided, basally widened, apically rounded, slightly pointed. In lateral view strongly bent.

**Female.** Length 7.8–8.4 mm, width 5.3–5.8 mm.

Antennae antennomeres 1–3 thin, antennomeres 7–11 wide and flat, with short hairs, antennomere 7, twice as long as wide; antennomeres 8–11 each approximately 1.6 × as long as wide; antennomere 2 shortest, antennomere 3 longer than 2 slightly, 1.5 × as long as second; antennomere 4 longest, slightly longer than antennomeres 2 and 3 combined; apical sternite flatted.

##### Differential diagnosis.

This species can be distinguished from other species by metallic green, red, or blue coloration of the body.

##### Distribution.

China: Henan, Zhejiang, Jiangxi, Hunan, Fujian, Taiwan, Guangdong, Guangxi, Sichuan, Guizhou, Xizang; Indo-China.

##### Host plant.

Rubiaceae.

**Figure 5.**
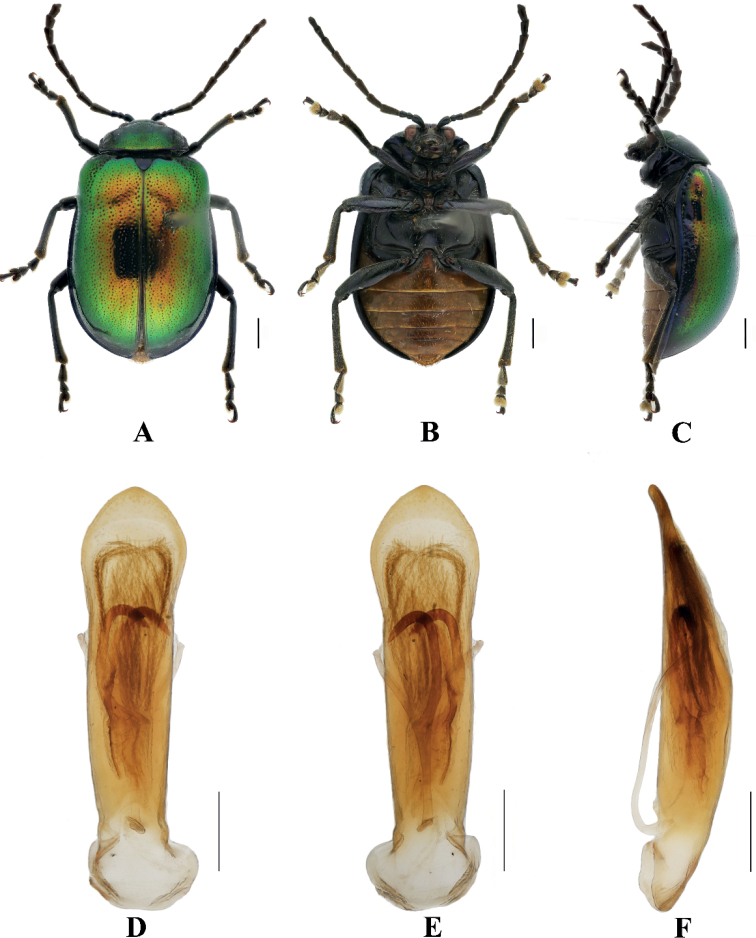
Sphenoraia (Sphenoraioides) micans**A–C** habitus **D–F** aedeagus **A, D** dorsal views **B, E** ventral views **C, F** lateral views. Scale bars: 1 mm (**A–C**); 0.5 mm (**D–F**).

#### ﻿ Sphenoraia (Sphenoraioides) nebulosa

Taxon classificationAnimaliaColeopteraChrysomelidae

(Gyllenhal, 1808)

BD499C53-C23C-5E2C-9E21-41B3117B19CF

Fig. 6A–F

Galleruca nebulosa Gyllenhal, 1808: 292.
Sphenoraia (Sphenoraioides) nebulosa : Laboissière 1934: 132.

##### Other specimens examined.

1♀, China, Hainan Province; 8 Aug. 1934; IZAS. 1♂, China, Hainan; 24 Mar. 1934; IZAS. 1♂, China, Hainan Province, Jianfengling; 13–17 Apr. 1984; IZAS. 1♂, Lingnan University; 10 May 1948; En-119989; SYSU. 1♂, China, Guangxi Province, Yangshuo; 29 Jun. 1938; IZAS. 1♂, China, Guangxi Province, Yangshuo; 6 Apr. 1938; IZAS. 1♀, China, Guangxi Province, Yangshuo; 14 Oct. 1938; IZAS. 1♀, China, Guangxi Province, Fulong, Pinglong Mountain; 650 m; 13 Mar. 1998; Gexia Qiao leg.; IZAS. 1♂, China, Guangxi Province, Longzhou, Nonggang; 330 m; 15 Jun. 2000; Wenzhu Li leg.; IZAS. 1♂, China, Guangxi Province, Guilin; 14 Aug. 1952; IZAS. 2♀, China, Guangxi Province, Guilin; 19 Sep. 1952; IZAS. 2♀, China, Guangxi Province, Guilin; 19 Sep. 1952; IZAS. 5♂5♀, China, Guangxi Province, Guilin; 6 Mar. 1952; IZAS. 1♀, China, Yunnan Province, Yiwubannan, Menglun; 650 m; 25 Jul. 1959; Yiran Zhang leg.; IZAS. 1♂, China, Yunnan Province, Yiwubannan, Menglun; 650 m; 3 Apr. 1964; Baolin Zhang leg.; 1♂, China, Yunnan Province, Xishuangbanna, Mengla; 620–650 m; 27 May 1959; Fuji Pu leg.; IZAS. 1♂, China, Yunnan Province, Xishuangbanna, Damenglong; 650 m; 13 Apr. 1958; Shuyong Wang leg.; IZAS.

##### Description.

**Male.** Length 6.2–6.8 mm, width 4.6–5.2 mm.

Head, pronotum, elytra and legs yellow, antennae and ventral surface of the body yellowish brown, scutellum brown; pronotum with a black spot on each side; each elytron with seven black spots, basal, middle, and subapical areas each with one pair of spots, apical area with one spot; some specimens have reduced or dark grey spots on the elytra, some have black spots interconnected.

Vertex finely and sparsely covered with punctures; frontal tubercle distinctly raised, separated from each other by a deep furrow; antennae short, robust, extend to the middle of the elytra; antennomeres 1–3 thin, shiny; antennomeres 4–11 wide and flat, with short hairs, antennomere 4 approximately 2.5 × as long as wide; antennomeres 5–10, each approximately 1.6 × as long as wide; antennomeres 2 and 3 shortest, antennomere 3 similar in length and shape to antennomere 2, antennomere 4 longest, 1.5 × as long as antennomeres 2 and 3 combined; antennomeres 5–10 gradually shortened, shorter than 4; antennomere 11 slightly longer than 10, pointed.

Pronotum approximately 2.5 × as wide as long, with rounded lateral margins; disc slightly convex, sparsely covered with small punctures, with the punctures on the pronotum larger than those on the head.

Scutellum triangular, smooth, impunctate.

Basal width of both elytra wider than the pronotum, gradually widen posteriorly and rounded at apex; dorsal surface slightly convex, irregularly covered with large and deep punctures, the interstices between punctures slightly wider than diameter of individual punctures.

Metasternum 2.5 × as long as mesosternum; prothoracic legs shortest, mesothoracic legs slightly longer, metathoracic legs longest.

Ventral surface of abdomen with five segments, segment 1 longest, segments 2–4 gradually shortened, apical segment slightly longer than segment 4, with three lobes.

Aedeagus slender, parallel-sided, basally widened, apex rounded. In lateral view moderately bent.

**Female.** Length 6.2–6.6 mm, width 4.5–5.0 mm.

Antennae yellow, antennomeres 6–11 brown; antennomeres 1–5 thin, antennomeres 6–11 wide and flat, each approximately 1.5 × as long as wide; antennomere 2 shortest, antennomere 3 slightly longer than 2, 1.2 × as long as second; antennomere 4 longest, slightly longer than antennomeres 2 and 3 combined; apical sternite flatted.

##### Differential diagnosis.

This species can be distinguished from the other species by black spots of the pronotum and elytra. However, it especially resembles Sphenoraia (Sphenoraioides) haizhuensis, the former differs in having a scutellum without punctures, and the aedeagus in lateral view being moderately bent.

##### Distribution:

China: Guangdong, Hainan, Guangxi, Yunnan; Vietnam, Laos, Cambodia, Thailand, Myanmar, India, Sikkim.

**Figure 6.**
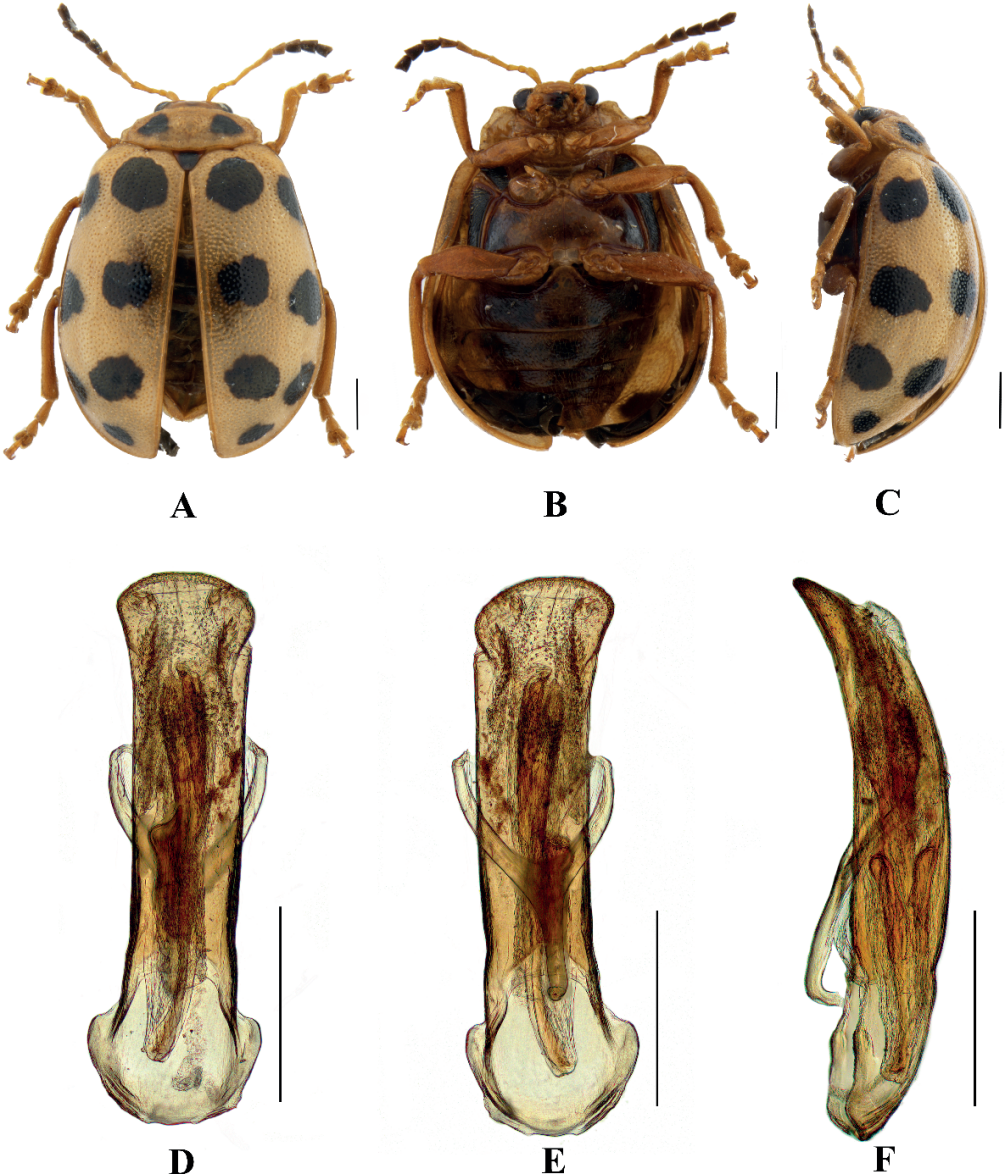
Sphenoraia (Sphenoraioides) nebulosa**A–C** habitus **D–F** aedeagus **A, D** dorsal views **B, E** ventral views **C, F** lateral views. Scale bars: 1 mm (**A–C**); 0.5 mm (**D–F**).

#### ﻿ Sphenoraia (Sphenoraioides) nigromaculata

Taxon classificationAnimaliaColeopteraChrysomelidae

Jiang, 1992

0A9F0D36-9552-58E0-9073-638DBC103CDC

Fig. 7A–F

Sphenoraia nigromaculata Jiang, 1992: 665.
Sphenoraia (Sphenoraioides) nigromaculata : Wang et al. 2000: 118.

##### Type specimen examined.

***Holotype***: ♂, China, Sichuan Province, Maerkang; 2500 m; 17 Aug. 1983; Shuyong Wang leg.; IZAS.

##### Additional specimen examined.

1♀, China, Sichuan Province, Xiaojin, Fubian; 2900 m; 19 Aug. 1963; Leyi Zheng leg.; IZAS.

##### Description.

**Male.** Length 6.0 mm, width 3.4 mm.

Head, pronotum and scutellum blackish green, antennae, legs, and ventral surface of body brown, elytra and apex of each abdominal segment yellow; each elytron with seven black spots of different sizes, basal, middle and subapex each with one pair of spots, apex with one spot.

Vertex densely covered with punctures; frontal tubercles distinctly raised, each separated from each other by a deep furrow; antennae short, robust, extending to the middle of the elytra; antennomere 2 shortest, antennomere 3 slightly longer than 2, 1.2 × as long as second; antennomere 4 longest, twice as long as antennomeres 2 and 3 combined; antennomeres 5–10 gradually shortened, shorter than 4; antennomere 11 slightly longer than 10, pointed.

Pronotum approximately 1.9 × as wide as long, with rounded lateral margins; disc slightly convex, sparsely covered in middle with small punctures with large punctures on other parts. The interstices between punctures slightly narrower than diameter of individual punctures and lightly covered with small punctures in interstices.

Scutellum triangular, densely covered with punctures.

Bases of both elytra wider than the pronotum, gradually widen posteriorly and rounded at apex; dorsal surface slightly convex, irregularly covered with large and deep punctures, the interstices between punctures slightly narrower than diameter of individual punctures and lightly covered with small punctures in interstices.

Metasternum twice as long as mesosternum; prothoracic legs shortest, mesothoracic legs slightly longer, metathoracic legs longest.

Ventral surface of abdomen with five segments, segment 1 longest, segments 2–4 gradually shortened, apical segment slightly longer than segment 3, three lobes.

Aedeagus slender, rounded laterally, basally widened, with triangular apex, slightly pointed. In lateral view moderately bent.

**Female.** Length 5.8 mm, width 3.3 mm.

Antennal antennomere 2 shortest, antennomere 3 slightly longer than 2, 1.4 × as long as second; apical sternite flatted.

##### Differential diagnosis.

This species can be distinguished from other species by the blackish green pronotum and blackish green spots of the elytra.

##### Distribution.

China: Sichuan.

**Figure 7.**
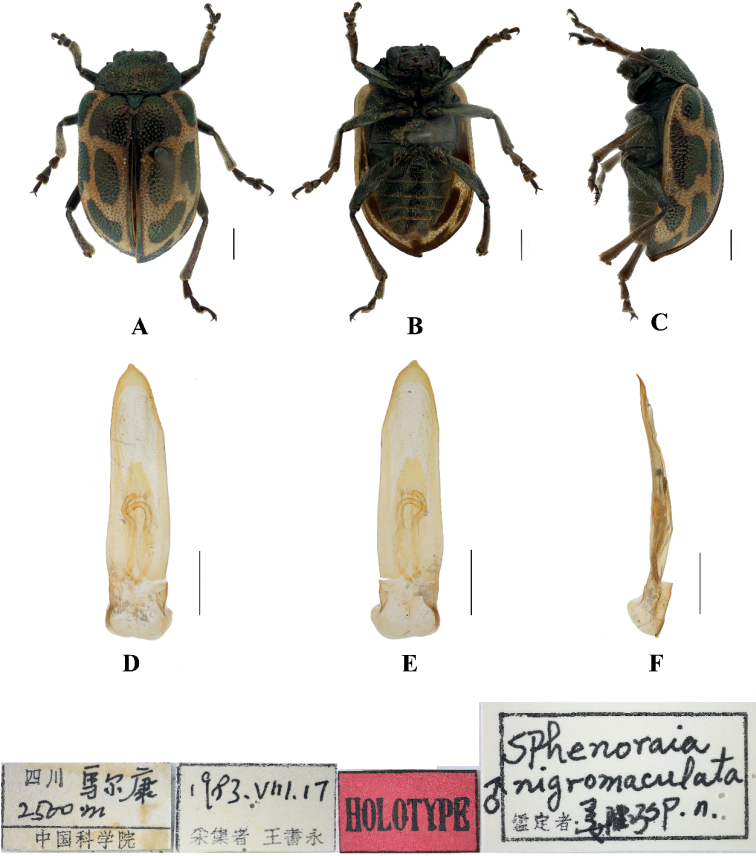
Sphenoraia (Sphenoraioides) nigromaculata**A–C** habitus **D–F** aedeagus **A, D** dorsal views **B, E** ventral views **C, F** lateral views. Scale bars: 1 mm (**A–C**); 0.5 mm (**D–F**).

#### ﻿ Sphenoraia (Sphenoraioides) punctipennis

Taxon classificationAnimaliaColeopteraChrysomelidae

Jiang, 1992

71DA49A1-E572-5EED-91E8-14CE8CDC98E4

Fig. 8A–F

Sphenoraia punctipennis Jiang, 1992: 665.
Sphenoraia (Sphenoraioides) punctipennis : Wang et al. 2000: 118.

##### Type specimen examined.

***Holotype***: ♂, China, Xizang, Mangkang, Haitong; 3250 m; Aug. 1982; Shuyong Wang leg.; IZAS.

##### Description.

**Male.** Length 6.0 mm, width 3.4 mm.

Head, pronotum and scutellum blackish green, antennae, elytra, legs, and ventral surface of body brown; elytral epipleuron from base to subapex yellow, with middle of suture yellow, connected by yellow stripes from the base to the apex of each elytron.

Vertex finely and sparsely covered with punctures; frontal tubercles distinctly raised, each separated by a deep furrow; antennae short, robust, extended to the middle of the elytra; antennomeres 1–3 thin, shiny; antennomeres 4–11 wide and flat, with short hairs, antennomeres 2 and 3 shortest, antennomere 3 similar in length and shape to antennomere 2, antennomere 4 longest, 1.5 × as long as antennomeres 2 and 3 combined; antennomeres 5–10 gradually shortened, shorter than 4; antennomere 11 slightly longer than 10, pointed.

Pronotum approximately 1.8 × as wide as long, with rounded lateral margins; disc slightly convex, sparsely covered with small punctures in the middle with large punctures on other parts. The interstices between punctures equal to the diameter of individual punctures and lightly covered with small punctures in interstices.

Scutellum triangular, sparsely covered with punctures at base.

Bases of both elytra wider than the pronotum, gradually widen posteriorly, and rounded at apex; dorsal surface slightly convex, irregularly covered with large and deep punctures, the interstices between punctures narrower than the diameter of individual punctures and lightly covered with small punctures in interstices, with their interstices somewhat wrinkled.

Metasternum twice as long as mesosternum; prothoracic legs shortest, mesothoracic legs slightly longer, metathoracic legs longest.

Ventral surface of abdomen with five segments, segment 1 longest, segments 2–4 gradually shortened, apical segment slightly longer than segment 4, three lobes.

Aedeagus slender, rounded laterally, basally widened, with triangular apex, distinctly pointed. In lateral view moderately bent.

##### Differential diagnosis.

This species can be distinguished from other species by blackish green pronotum, yellow stripes of elytra and large punctures on elytra.

##### Distribution.

China: Xizang.

##### Host plant.

*Rheum* sp.

**Figure 8.**
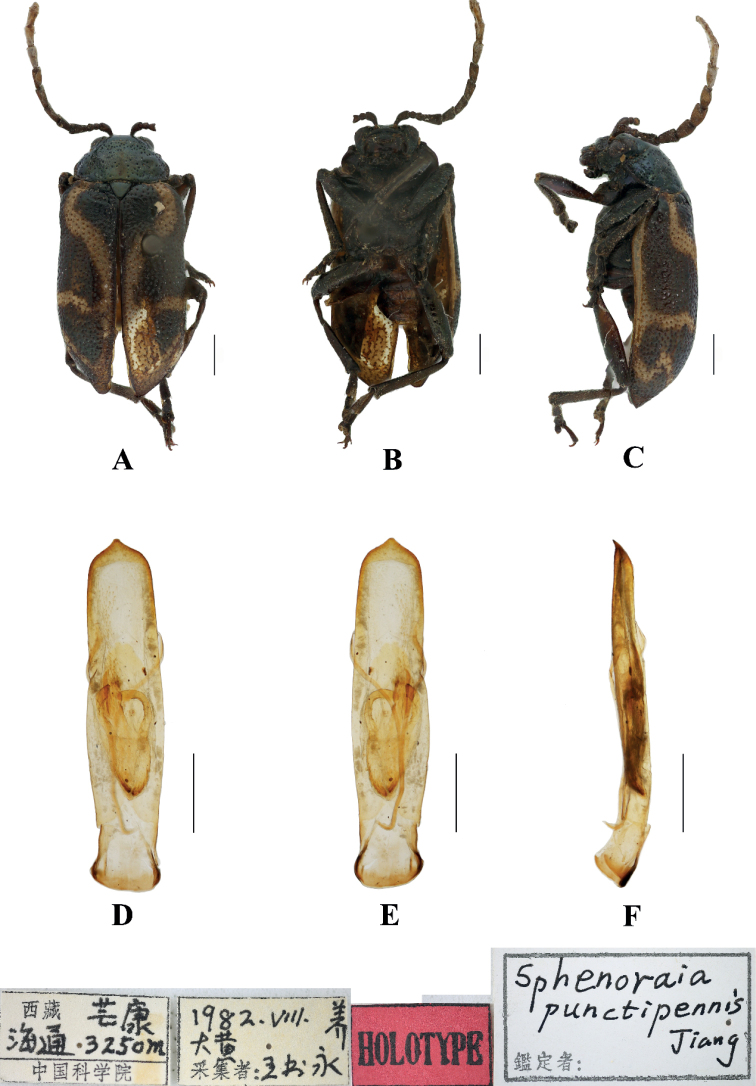
Sphenoraia (Sphenoraioides) punctipennis**A–C** habitus **D–F** aedeagus **A, D** dorsal views **B, E** ventral views **C, F** lateral views. Scale bars: 1 mm (**A–C**); 0.5 mm (**D–F**).

#### ﻿ Sphenoraia (Sphenoraioides) rutilans

Taxon classificationAnimaliaColeopteraChrysomelidae

(Hope, 1831)

F7A940F1-8CE9-5CED-BF46-BB20AAD061EC

Fig. 9A–F

Eumolpus rutilans Hope, 1831: 30.
Chrysomela mutabilis Hope, 1831: 30. Synonymized by Maulik 1936: 547.
Galleruca fulgida Kollar & Redtenbacher, 1844: 554. Synonymized by Maulik 1936: 547.
Sphenoraia cyanea Allard, 1890: 92. Synonymized by Laboissière 1940: 30.
Sphenoraia (Sphenoraioides) rutilans : Gressitt and Kimoto 1963: 657.

##### Other specimens examined.

1♂, China, Yunnan Province, Xishuangbanna, Menglun; 600 m; 22 Apr. 1994; Longlong Yang leg.; IZAS. 1♂, China, Yunnan Province, Xishuangbanna, Mengla; 620–650 m; 2 May 1959; Facai Zhang leg.; IZAS. 1♂, China, Yunnan Province, Xishuangbanna, Mengla; 620–650 m; 3 May 1959; Facai Zhang leg.; IZAS. 1♂, China, Yunnan Province, Xishuangbanna, Mengla; 620–650 m; 3 May 1959; Facai Zhang leg.; IZAS. 1♀, China, Yunnan Province, Xishuangbanna, Mengla; 800 m; 1 Jun. 1958; Shuyong Wang leg.; IZAS. 2♂♂1♀, China, Yunnan Province, Xishuangbanna, Menghun; 1200–1400 m; 3 Jun. 1958; Shuyong Wang leg.; IZAS. 1♂, China, Yunnan Province, Yiwubannan, Menglun; 650 m; 3 Aug. 1959; Yiran Zhang leg.; IZAS. 1♀, Yunnan Province, Xishuangbanna,Damenglong; 650 m; 5 Oct. 1958; Zhizi Chen leg.; IZAS. 1♀, Yunnan Province, Xishuangbanna, Damenglong; 650 m; 7 Oct. 1958; Zhizi Chen leg.; IZAS.

##### Description.

**Male.** Length 7.8–8.2 mm, width 4.9–5.2 mm.

Body dark blue, antennae brown.

Vertex finely and sparsely covered with punctures; frontal tubercles distinctly raised, each separated from each other by a deep furrow; antennae short, robust, extended to the middle of the elytra; antennomeres 1–3 thin, shiny; antennomeres 4–11 wide and flat, with short hairs, antennomere 4 approximately 1.5 × as long as wide; antennomeres 5–6, each approximately 1.2 × as long as wide; the length of each of antennomeres 7–9 equals its width; antennomere 10 approximately 1.2 × as wide as long; antennomere 11 approximately 1.2 × as long as wide; antennomeres 2 and 3 shortest, antennomere 3 similar in length and shape to antennomere 2, antennomere 4 longest, 1.2 × as long as antennomeres 2 and 3 combined; antennomeres 5–10 gradually shortened, shorter than 4; antennomere 11 slightly longer than 10, pointed.

Pronotum approximately twice as wide as long, with rounded lateral margins; disc sparsely covered with punctures, with a lateral pair of shallow impressions.

Scutellum triangular, with rounded apex, smooth, impunctate.

Bases of both elytra wider than the pronotum, gradually widen posteriorly and rounded at apex; dorsal surface slightly convex, irregularly covered with punctures, the interstices between punctures equal to diameter of individual punctures.

Metasternum twice as long as mesosternum; prothoracic legs shortest, mesothoracic legs slightly longer, metathoracic legs longest.

Ventral surface of abdomen with 5 segments, segment 1 longest, segments 2–4 gradually shortened, apical segment slightly longer than segment 3, three lobes.

Aedeagus slender, parallel-sided, basally widened, apex rounded. In lateral view strongly bent.

**Female.** Length 8.0–8.2 mm, width 5.0–5.4 mm.

Antennae antennomeres 1–3 thin, shiny; antennomeres 4–11 with short hairs, antennomeres 7–11 wide and flat, each approximately 1.2 × as wide as long; apical sternite flatted.

##### Differential diagnosis.

This species can be distinguished from other species by wide and flat antennae and the shallow impressions of the pronotum.

##### Distribution.

China: Yunnan; Kashmir, Myanmar, India, Nepal, Bhutan, Bangladesh, Pakistan.

**Figure 9.**
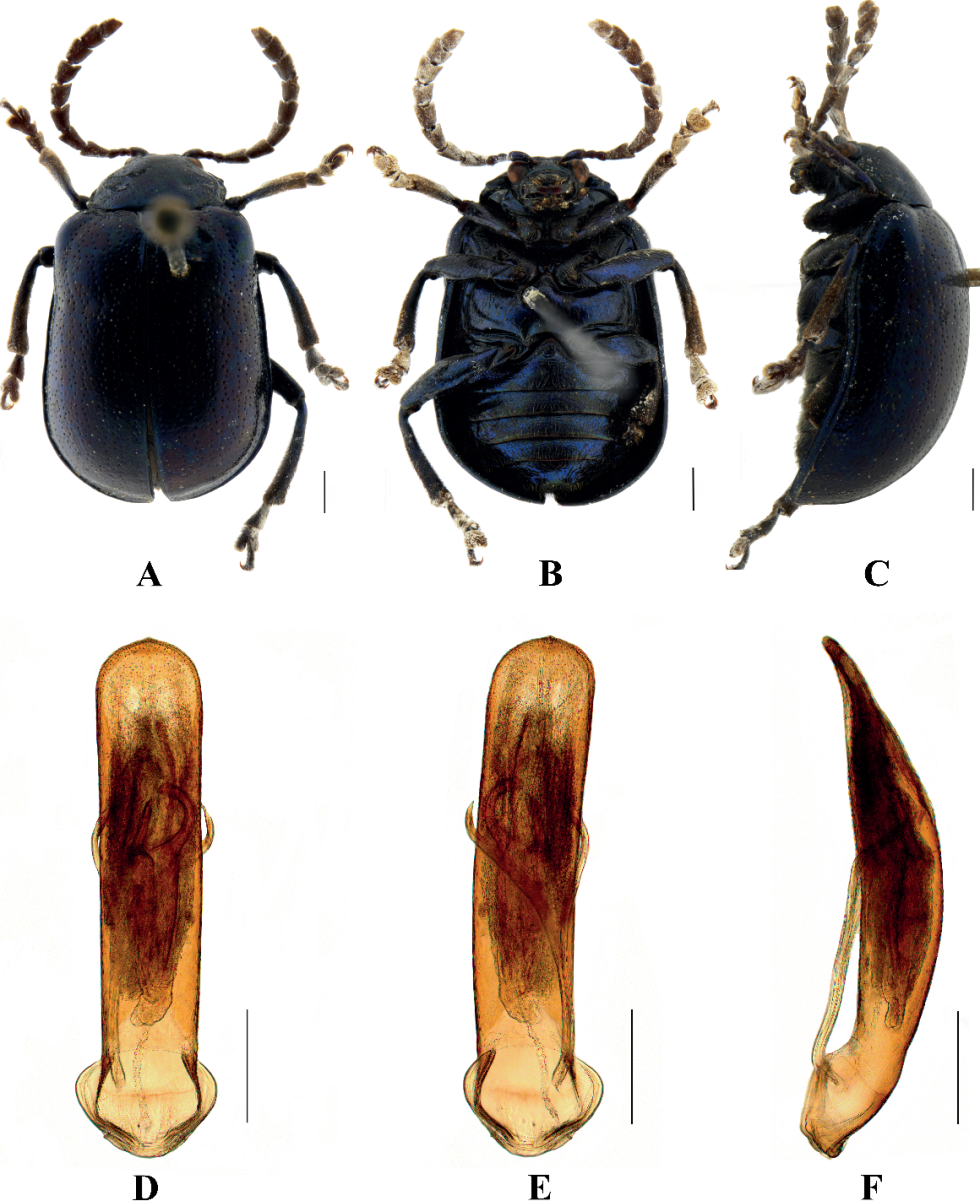
Sphenoraia (Sphenoraioides) rutilans**A–C** habitus **D–F** aedeagus **A, D** dorsal views **B, E** ventral views **C, F** lateral views. Scale bars: 1 mm (**A–C**); 0.5 mm (**D–F**).

#### ﻿ Sphenoraia (Sphenoraioides) yajiangensis

Taxon classificationAnimaliaColeopteraChrysomelidae

Jiang, 1992

BE4EDFF4-6C3C-5CE7-BA82-8A4BE28FAA2B

Fig. 10A–F

Sphenoraia yajiangensis Jiang, 1992: 667.
Sphenoraia (Sphenoraioides) yajiangensis : Wang et al. 2000: 118.

##### Type specimens examined.

***Holotype***: ♂, China, SiChuan Province, YaJiang; 3600 m; 26 Aug. 1982; Shuyong Wang leg.; IZAS. ***Paratypes***: 3♂♂1♀, same information as holotype. ***Allotype***: 1♀, same information as holotype.

##### Description.

**Male.** Length 5.8–6.2 mm, width 3.6–4.0 mm.

Head, antennae, and legs brown, pronotum, scutellum and elytra blackish green, ventral surface of body black, elytral epipleuron from base to apical 1/3 and apex of each abdominal segment yellow.

Vertex finely and sparsely covered with punctures; frontal tubercles distinctly raised, separated from each other by a deep furrow; antennae short, robust, extended to the middle of elytra; antennomeres 1–3 thin, shiny; antennomeres 4–11 wide and flat, with short hairs, antennomere 4 approximately twice as long as wide; antennomeres 5–10, each approximately 1.5 × as long as wide; antennomere 2 shortest, antennomere 3 slightly longer than 2, 1.2 × as long as second; antennomere 4 longest, 1.7 × as long as antennomeres 2 and 3 combined; antennomeres 5–10 gradually shortened, shorter than 4; antennomere 11 slightly longer than 10, pointed.

Pronotum approximately twice as wide as long, with rounded lateral margins, disc slightly convex, sparsely covered in middle with small punctures with large punctures on other parts. The interstices between punctures equal to the diameter of individual punctures and lightly covered with small punctures in interstices.

Scutellum triangular, with rounded apex, covered with small punctures at base.

Bases of both elytra wider than pronotum, gradually widen posteriorly and rounded at apex; dorsal surface slightly convex, irregularly covered with large and deep punctures, the interstices between punctures equal to the diameter of individual punctures and lightly covered with small punctures in interstices.

Metasternum twice as long as mesosternum; prothoracic legs shortest, mesothoracic legs slightly longer, metathoracic legs longest.

Ventral surface of abdomen with five segments, segment 1 longest, segments 2–4 gradually shortened, apical segment slightly longer than segment 4, three lobes.

Aedeagus slender, parallel-sided, basally widened, apex narrowly pointed; in lateral view moderately bent.

**Female**. Length 5.8–6.3 mm, width 3.5–3.9 mm.

Antennal antennomeres 4–11 thin, antennomere 2 shortest, antennomere 3 slightly longer than 2, 1.5 × as long as second; antennomere 4 longest, 1.2 × as long as antennomeres 2 and 3 combined; apical sternite flatted.

##### Differential diagnosis.

This species can be distinguished from other species by blackish green pronotum and elytra.

##### Distribution.

China: Sichuan.

##### Host plant.

Berberidaceae.

**Figure 10.**
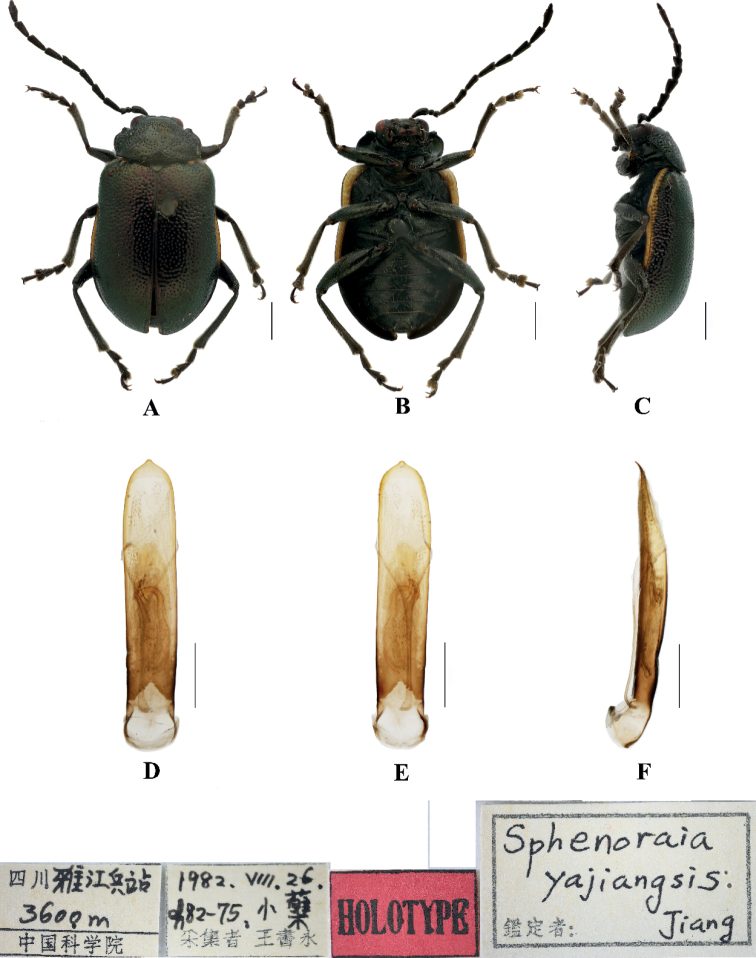
Sphenoraia (Sphenoraioides) yajiangensis**A–C** habitus **D–F** aedeagus **A, D** dorsal views **B, E** ventral views **C, F** lateral views. Scale bars: 1 mm (**A–C**); 0.5 mm (**D–F**).

#### ﻿ Sphenoraia (Sphenoraia) decemmaculata sp. nov.

Taxon classificationAnimaliaColeopteraChrysomelidae

7DEFAC93-B5EB-58F3-8441-1B4B3EECCA03

https://zoobank.org/9C8B84E5-C6E1-4FE1-91EF-A1D4304CAEA3

Fig. 11A–F

##### Type specimens examined.

***Holotype***: ♂, China, Yunnan Province, E’shan; Aug. 1980; IZAS. ***Paratype***: 1♀, China, Sichuan Province, Guichang, liusuo; 10 Jun. 1961; Dingxi Lao leg.; IZAS.

##### Description.

**Male.** Length 6.2 mm, width 4.5 mm.

Head and pronotum yellowish brown, antennae, scutellum, ventral surface of body and legs black, elytra yellow, each with five black spots, basal and middle areas of each elytron with one pair of spots, subapical area with one spot.

Vertex finely and sparsely covered with punctures; frontal tubercles distinctly raised, separated from each other by a deep furrow; antennae slender, extended to the middle of the elytra; antennomeres 1–3 thin, shiny; antennomeres 4–11 with short hair, antennomere 4 approximately twice as long as wide; antennomeres 5–10, each approximately 1.6 × as long as wide; antennomere 2 shortest, antennomere 3 slightly longer than 2, 1.5 × as long as second; antennomere 4 longest, 1.2 × as long as antennomeres 2 and 3 combined; antennomeres 5–10 gradually shortened, shorter than 4; antennomere 11 slightly longer than 10, pointed.

Pronotum approximately twice as wide as long, with lateral margins straight and parallel, anterior angle thickened, protruding forwards, disc slightly convex and sparsely covered with small punctures.

Scutellum triangular, sparsely covered with small punctures.

Bases of both elytra wider than pronotum, gradually widen posteriorly and rounded at apex; dorsal surface slightly convex and irregularly covered with large, deep punctures, the interstices between punctures slightly wider than the diameter of individual punctures.

Metasternum 2.5 × as long as mesosternum; prothoracic legs shortest, mesothoracic legs slightly longer, metathoracic legs longest.

Ventral surface of abdomen with 5 segments, segment 1 longest, segments 2–4 gradually shortened, apical segment slightly longer than segment 4, three lobes.

Aedeagus short and wide, parallel-sided, gradually widened apically and rounded at apex, basally widened; in lateral view strongly bent.

**Female.** Length 6.3 mm, width 4.4 mm.

Antennae slender, antennomeres 4–11 thin, with short hairs, antennomere 2 shortest, antennomere 3 slightly longer than 2, 1.2 × as long as second; apical sternite flatted.

##### Differential diagnosis.

The new species closely resembles Sphenoraia (Sphenoraioides) anjiensis but differs due to the black pronotum and yellow abdomen. In the new species the head and pronotum are brown, and each elytron has five black spots: the base and middle of each elytron with a pair of spots, the subapex with one spot. The aedeagus is short and wide, gradually widening apically and is rounded at the apex.

##### Etymology.

Latin: *deca* = ten; *macula* = spot; referring to the ten black spots on the elytra.

##### Distribution.

China: Sichuan, Yunnan.

**Figure 11.**
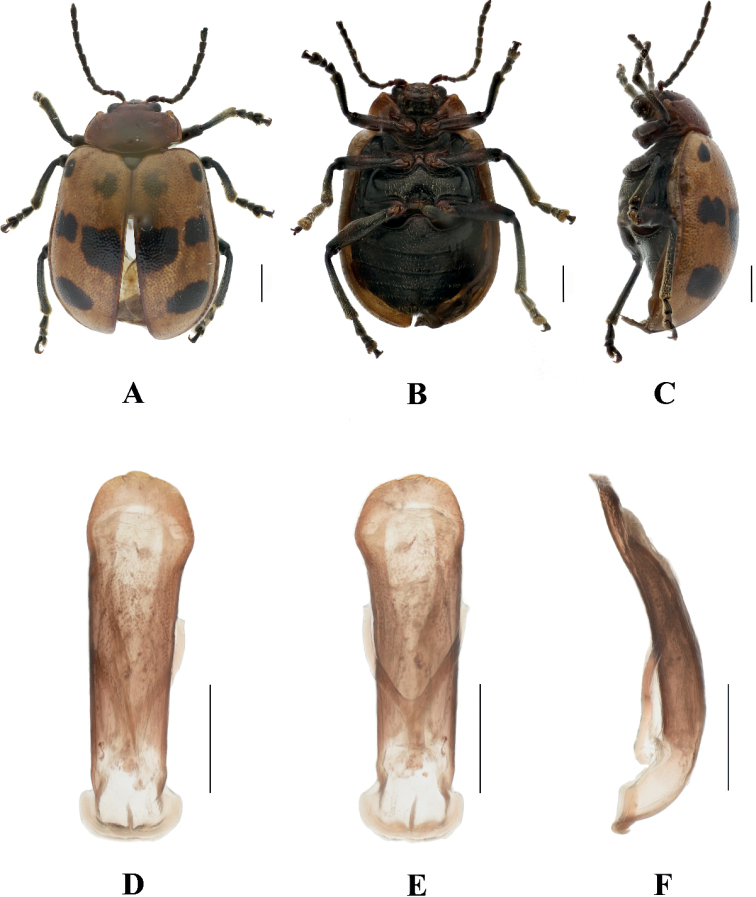
Sphenoraia (Sphenoraia) decemmaculata sp. nov. **A–C** habitus **D–F** aedeagus **A, D** dorsal views **B, E** ventral views **C, F** lateral views. Scale bars: 1 mm (**A–C**); 0.5 mm (**D–F**).

#### ﻿ Sphenoraia (Sphenoraioides) flavomarginata sp. nov.

Taxon classificationAnimaliaColeopteraChrysomelidae

E0DC6051-8427-5465-8671-8FF4980E15B8

https://zoobank.org/C0361BA0-9E50-42DB-8005-50EA1087710A

Fig. 12A–F

##### Type specimens examined.

***Holotype***: ♂, China, Sichuan Province, Kangding, liuba; 3700 m; Sep. 1982; S.Y. Wang leg.; IZAS. ***Paratype***: 3♀♀, same data as holotype.

##### Description.

**Male.** Length: 5.5–6.0 mm, width: 2.8–3.5 mm.

Antennae, ventral surface of the body, and legs brown. Head, pronotum, scutellum, and elytra blackish green, apical area of each segment of the abdomen yellow, elytra with yellow stripes along the basal margin, extending along the elytral epipleuron from the base to the apical 1/3, with one transverse yellow stripe at subapex.

Vertex finely and sparsely covered with punctures; frontal tubercles distinctly raised, separated from each other by a deep furrow; antennae short, robust, extend to the middle of the elytra; antennomeres 1–3 thin, shiny; antennomeres 4–11 wide and flat, with short hairs, antennomere 4 approximately 3 × as long as wide; antennomeres 5–10, each approximately 2 × as long as wide; antennomere 2 shortest, antennomere 3 slightly longer than 2, 1.2 × as long as second; antennomere 4 longest, 1.5 × as long as antennomeres 2 and 3 combined; antennomeres 5–10 gradually shortened, shorter than 4; antennomere 11 slightly longer than 10, pointed.

Pronotum approximately twice as wide as long, with rounded lateral margins; disc slightly convex, sparsely covered with small punctures in the middle with large punctures on other parts of pronotum. The interstices of punctures equal to diameter of punctures slightly, covered with small punctures.

Scutellum triangular, with rounded apex, covered with small punctures and short hairs.

Bases of both elytra wider than pronotum, gradually widen posteriorly and rounded at apex; dorsal surface slightly convex, irregularly covered with large and deep punctures, the interstices between punctures equal to the diameter of individual punctures and lightly covered with small punctures in interstices.

Metasternum twice as long as mesosternum; prothoracic legs shortest, mesothoracic legs slightly longer, metathoracic legs longest.

Ventral surface of abdomen with 5 segments, segment 1 longest, segments 2–4 gradually shortened, apical segment slightly longer than segment 4, three lobes.

Aedeagus slender, parallel-sided, basally widened, apex narrowly pointed; in lateral view moderately bent.

**Female.** Length: 5.4–6.0 mm, width: 2.9–3.6 mm.

Antennae slender, antennomeres 4–11 thin, antennomere 2 shortest, antennomere 3 slightly longer than 2, 1.2 × as long as second; apical sternite flatted.

##### Differential diagnosis.

The new species closely resembles Sphenoraia (Sphenoraioides) yajiangensis. However, the new species has a different pattern in the arrangement of the yellow stripes, with one transverse yellow stripe present at the subapex of the elytra, and the pronotum has sparse punctures. The aedeagus is slender, and its apex narrowly pointed.

##### Etymology.

Latin: *flava* = yellow; *margin* = margin; referring to each elytron with a yellow margin.

##### Distribution.

China: Sichuan.

**Figure 12.**
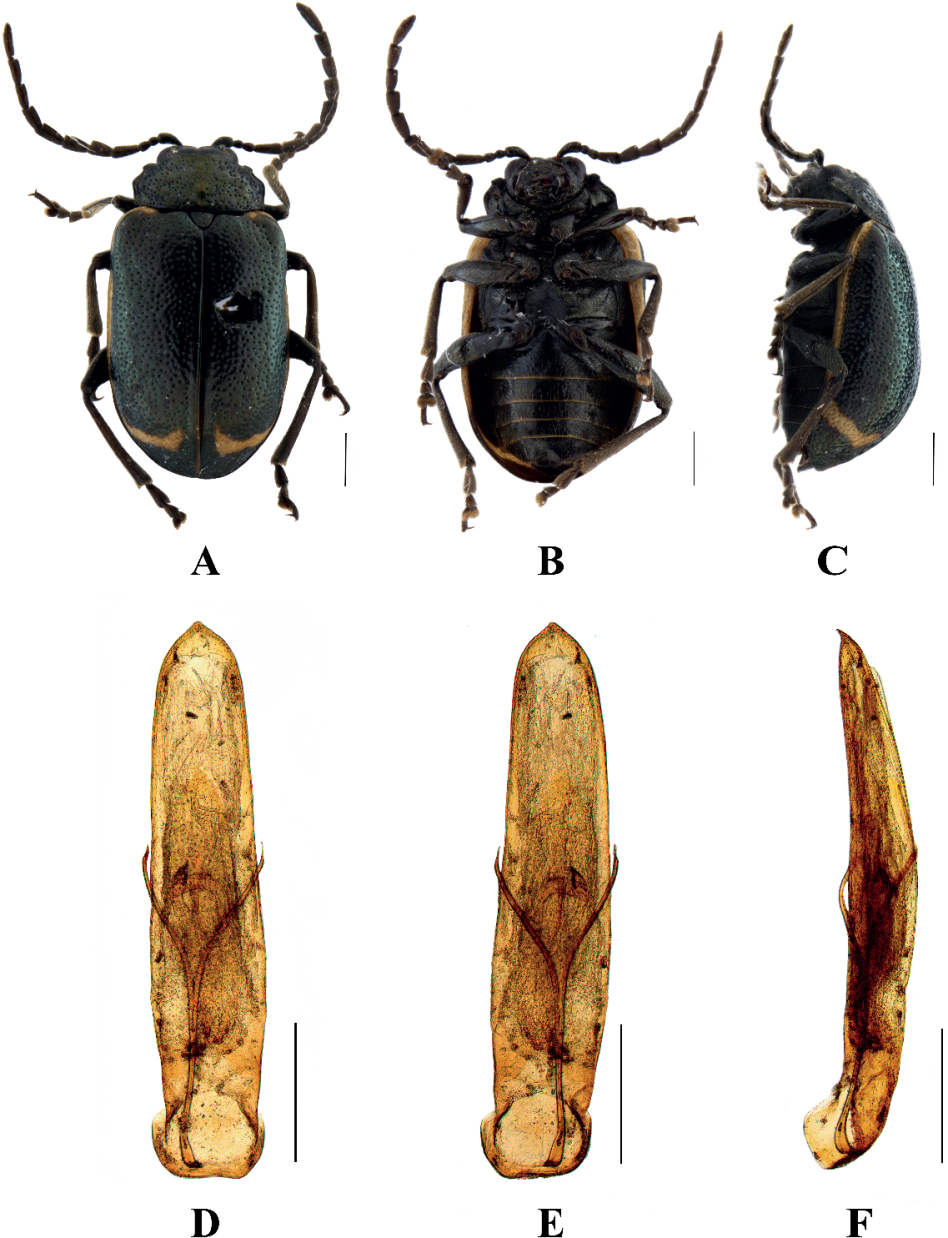
Sphenoraia (Sphenoraioides) flavomarginata sp. nov. **A–C** habitus **D–F** aedeagus **A, D** dorsal views **B, E** ventral views **C, F** lateral views. Scale bars: 1 mm (**A–C**); 0.5 mm (**D–F**).

#### ﻿ Gallerucida cupreata

Taxon classificationAnimaliaColeopteraChrysomelidae

(Jacoby, 1890) comb. nov.

18F13FD4-D3F1-56E3-9859-857E7C374A0A

Fig. 13 A–C

Sphenoraia cupreata Jacoby, 1890, 23: 193.

##### Type specimen examined.

Chang Yang, A. E. Pratt Coll., June 1888; 1^st^ Jacoby Coll.; Type 18239; *S.cupreata* Jac.

##### Description.

**Male.** Length 4.4 mm, width 3.2 mm

Head, pronotum, elytra, and scutellum green, antennae and legs brown, ventral surface of body yellowish brown.

Vertex finely and sparsely covered with punctures; frontal tubercle distinctly raised, separated from each other by a deep furrow; antennae short, robust, extended to the middle of elytra; antennomeres 1–3 thin, shiny; antennomeres 4–11 wide and flat, with short hairs, antennomeres 2 and 3 shortest, antennomere 3 similar in length and shape to antennomere 2, antennomere 4 longest, 1.2 × as long as antennomeres 2 and 3 combined; antennomeres 5–10 differ in length, shorter than 4; antennomere 11 slightly longer than 10, pointed.

Pronotum approximately 2.5 × as wide as long, with rounded lateral margins; disc sparsely covered with punctures, with a lateral pair of shallow impressions.

Scutellum triangular, covered with small punctures.

Bases of both elytra wider than pronotum, gradually widen posteriorly and rounded at apex; dorsal surface slightly convex, irregularly covered with large and deep punctures, the interstices between punctures slightly wider than the diameter of individual punctures and covered with small punctures.

Metasternum 2.5 × as long as mesosternum, Anterior metasternal process extending beyond the front edge of the meso-coxal cavities; prothoracic legs shortest, mesothoracic legs slightly longer, metathoracic legs longest.

Ventral surface of abdomen with five segments, segment 1 longest, segments 2–4 gradually shortened, apical segment equal in length to segment 1, with three lobes.

##### Notes.

According to the characteristics of the cylindrical process of the metasternum, Sphenoraia (Sphenoraia) cupreata is transferred from *Sphenoraia* to *Gallerucida*.

##### Distribution.

China: Hubei.

**Figure 13.**
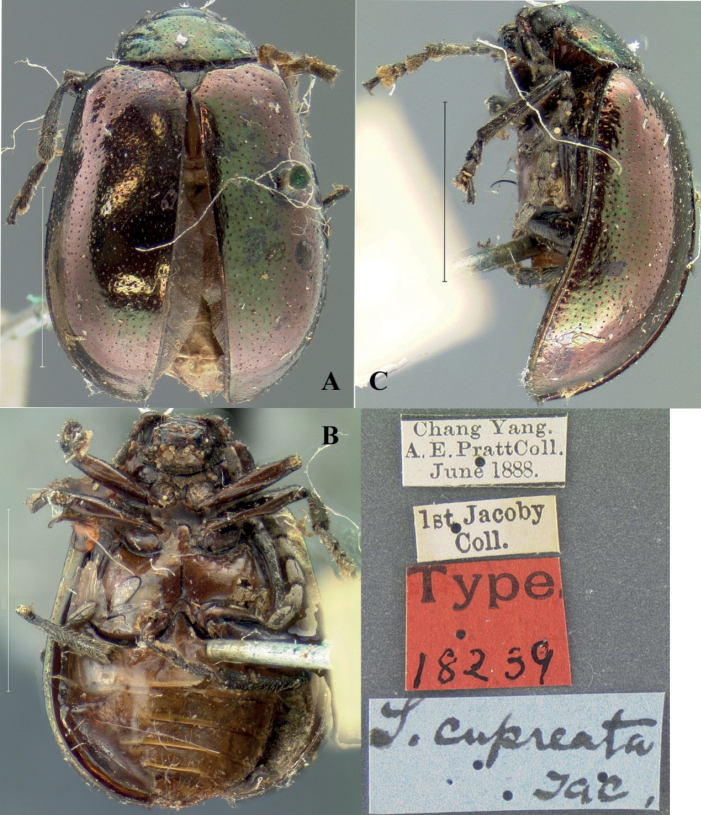
*Gallerucidacupreata*. comb. nov. **A–C** habitus **A** dorsal view **B** ventral view **C** lateral view. Scale bars: 2 mm.

#### ﻿ Gallerucida nigra

Taxon classificationAnimaliaColeopteraChrysomelidae

(Wang, Li & Yang, 2000) comb. nov.

CA2A17C4-EC1E-5903-B3BA-FAD219A64A20

Fig. 14A–C

Sphenoraia (Sphenoraia) nigra Wang, Li & Yang, 2000: 118.

##### Type specimens examined.

***Holotype***: ♀, China, Gansu Province, Dangchang; 1700–2300 m; 9 Jul. 1998; Shuyong Wang leg.; IZAS. ***Paratype***: 1♀, China, Gansu Province, Wen Country; 1400 m; 2 Jun. 1992; Hongjian Wang leg.; IZAS. 1♀, China, Gansu Province, Zhouqu; 2350 m; 5 Jul. 1998; Jun Chen; IZAS.

##### Other specimens examined.

1♀, China, Henan, Baiyun Mountain; 1900 m; 23. Jul. 2002; Lijie Zhang leg.; IZAS.

##### Description.

**Female.** Length: 6.6–6.8 mm, width: 4.0–4.5 mm.

Head, antennae, pronotum, scutellum, ventral surface of the body, and legs black, elytra brown; each elytron with eight black spots, basal and middle sections with one pair of spots, subapical area with three spots and apical area with one spot.

Vertex finely and sparsely covered with punctures; frontal tubercles distinctly raised, separated from each other by a deep furrow; antennae short, robust, extend to the middle of the elytra; antennomeres 1–3 thin, shiny; antennomeres 4–11 wide and flat, with short hairs, each approximately 2.5 × as long as wide; antennomere 2 shortest, antennomere 3 slightly longer than 2, 1.5 × as long as second; antennomere 4 longest, 1.2 × as long as antennomeres 2 and 3 combined; antennomeres 5–10 unequal in length, shorter than 4; antennomere 11 slightly longer than 10, pointed.

Pronotum approximately 1.8 × as wide as long, with rounded lateral margins; disc slightly convex, sparsely covered in middle with small punctures with large punctures laterally.

Scutellum triangular, smooth, impunctate.

Bases of both elytra wider than pronotum, gradually widen posteriorly and rounded at apex; dorsal surface slightly convex, irregularly covered with large and deep punctures, the interstices between punctures slightly wider than the diameter of individual punctures and covered with small punctures.

Metasternum 2.5 × as long as mesosternum, Anterior metasternal process extending beyond the front edge of the meso-coxal cavities; prothoracic legs shortest, mesothoracic legs slightly longer, metathoracic legs longest.

##### Notes.

According to the anterior metasternal process clearly extending beyond the front edge of the meso-coxal cavities, Sphenoraia (Sphenoraia) nigra is transferred from *Sphenoraia* to *Gallerucida*.

##### Distribution.

China: Henan, Gansu.

**Figure 14.**
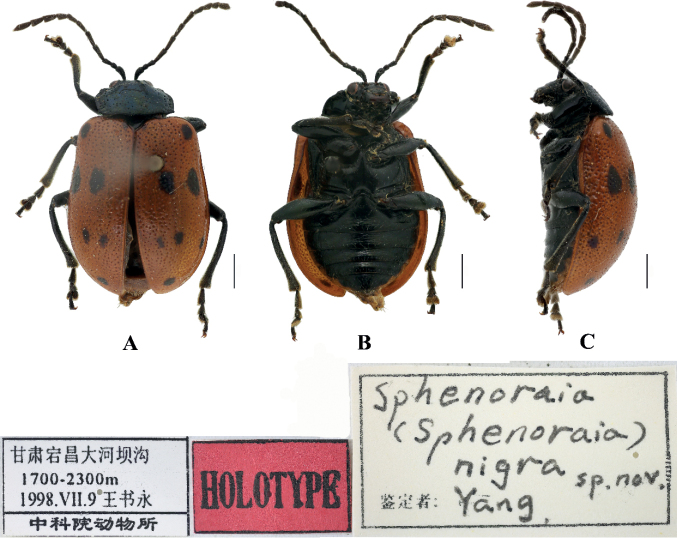
*Gallerucidanigra*. comb. nov. **A–C** habitus **A** dorsal view **B** ventral view **C** lateral view. Scale bars: 1 mm.

## Supplementary Material

XML Treatment for Sphenoraia

XML Treatment for Sphenoraia (Sphenoraioides) anjiensis

XML Treatment for Sphenoraia (Sphenoraioides) berberii

XML Treatment for Sphenoraia (Sphenoraioides) duvivieri

XML Treatment for Sphenoraia (Sphenoraioides) haizhuensis

XML Treatment for Sphenoraia (Sphenoraioides) micans

XML Treatment for Sphenoraia (Sphenoraioides) nebulosa

XML Treatment for Sphenoraia (Sphenoraioides) nigromaculata

XML Treatment for Sphenoraia (Sphenoraioides) punctipennis

XML Treatment for Sphenoraia (Sphenoraioides) rutilans

XML Treatment for Sphenoraia (Sphenoraioides) yajiangensis

XML Treatment for Sphenoraia (Sphenoraia) decemmaculata

XML Treatment for Sphenoraia (Sphenoraioides) flavomarginata

XML Treatment for Gallerucida cupreata

XML Treatment for Gallerucida nigra
